# *catena*-Poly[[tetra­aqua­[*trans*-1,2-bis­(4-pyrid­yl)ethene-κ^2^ *N*:*N*′]nickel(II)] dinitrate]

**DOI:** 10.1107/S1600536811007021

**Published:** 2011-03-02

**Authors:** Min Young Hyun, Pan-Gi Kim, Cheal Kim, Youngmee Kim

**Affiliations:** a Department of Fine Chemistry, Seoul National University of Science and Technology, Seoul 139-743, Republic of Korea; b Department of Forest & Environment Resources, Kyungpook National University, Sangju 742-711, Republic of Korea; c Department of Chemistry and Nano Science, Ewha Womans University, Seoul 120-750, Republic of Korea

## Abstract

In the title compound, {[Ni(C_12_H_10_N_2_)(H_2_O)_4_](NO_3_)_2_}_*n*_, the Ni^II^ ion, lying on a crystallographic inversion center, has a distorted octa­hedral coordination sphere comprising four water ligands and two N-atom donors from the *trans*-related 1,2-bis­(4-pyrid­yl)ethene ligands, which also have crystallographic inversion symmetry. These ligands bridge the Ni^II^ complex units, forming chains extending along the [110] and [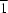
10] directions. The nitrate counter-anions stabilize the crystal structure through water–nitrate O—H⋯O hydrogen bonds.

## Related literature

For inter­actions of metal ions with amino acids, see: Daniele *et al.* (2008 ▶); Parkin (2004 ▶); Tshuva & Lippard (2004 ▶). For related complexes,see: Lee *et al.* (2008 ▶); Yu *et al.* (2008 ▶); Park *et al.* (2008 ▶); Shin *et al.* (2009 ▶); Yu *et al.* (2009 ▶, 2010 ▶); Kim *et al.* (2011 ▶).
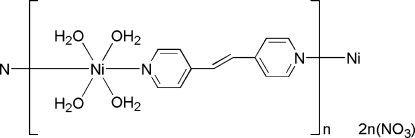


## Experimental

### 

#### Crystal data

[Ni(C_12_H_10_N_2_)(H_2_O)_4_](NO_3_)_2_
*M* *_r_* = 436.99
Monoclinic, 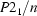

*a* = 7.415 (3) Å
*b* = 11.426 (4) Å
*c* = 10.950 (4) Å
β = 97.307 (7)°
*V* = 920.1 (6) Å^3^
*Z* = 2
Mo *K*α radiation
μ = 1.11 mm^−1^
*T* = 293 K
0.15 × 0.08 × 0.08 mm

#### Data collection

Bruker SMART CCD area-detector diffractometer
4954 measured reflections
1799 independent reflections
1116 reflections with *I* > 2σ(*I*)
*R*_int_ = 0.173

#### Refinement

*R*[*F* ^2^ > 2σ(*F* ^2^)] = 0.068
*wR*(*F* ^2^) = 0.238
*S* = 1.14
1799 reflections
136 parameters
4 restraints
H atoms treated by a mixture of independent and constrained refinement
Δρ_max_ = 1.08 e Å^−3^
Δρ_min_ = −1.86 e Å^−3^

### 

Data collection: *SMART* (Bruker, 1997 ▶); cell refinement: *SAINT* (Bruker, 1997 ▶); data reduction: *SAINT*; program(s) used to solve structure: *SHELXS97* (Sheldrick, 2008 ▶); program(s) used to refine structure: *SHELXL97* (Sheldrick, 2008 ▶); molecular graphics: *SHELXTL* (Sheldrick, 2008 ▶); software used to prepare material for publication: *SHELXTL*.

## Supplementary Material

Crystal structure: contains datablocks I, global. DOI: 10.1107/S1600536811007021/zs2096sup1.cif

Structure factors: contains datablocks I. DOI: 10.1107/S1600536811007021/zs2096Isup2.hkl

Additional supplementary materials:  crystallographic information; 3D view; checkCIF report

## Figures and Tables

**Table 1:** Hydrogen-bond geometry (Å, °)

*D*—H⋯*A*	*D*—H	H⋯*A*	*D*⋯*A*	*D*—H⋯*A*
O2—H2*B*⋯O3^i^	0.93 (7)	2.28 (8)	3.176 (9)	162 (8)
O2—H2*A*⋯O5^ii^	0.93 (6)	2.14 (7)	3.068 (8)	176 (7)
O1—H1*B*⋯O3^iii^	0.93 (4)	2.29 (2)	3.212 (9)	170 (8)
O1—H1*A*⋯O4^iv^	0.93 (6)	2.37 (3)	3.252 (8)	158 (7)

Symmetry codes: (i) 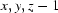; (ii) 
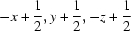
; (iii) 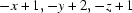; (iv) 
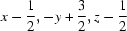
.

**Fig. 1.**
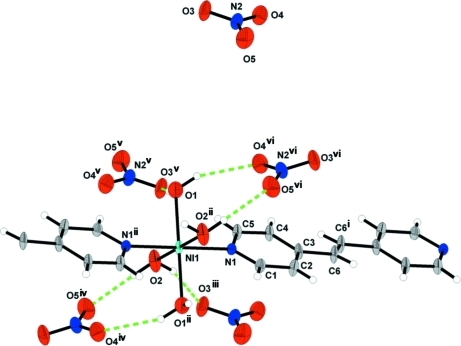
A fragment of one-dimensional chain structure of the title compound showing the atom numbering scheme. Displacement ellipsoids are drawn at the 30% probability level. Inter-species hydrogen bonds are shown as dashed lines. For symmetry codes: (i) (-x + 1, -y + 1, -z); (ii) -x, -y + 2, -z). For other codes, see Table 1.

## supplementary crystallographic information

### Comment

The interaction of transition metal ions with biologically active molecules such as amino acids and various acids is very important in biological systems (Daniele *et al.*, 2008; Parkin, 2004; Tshuva & Lippard, 2004). In attempting to model the interaction, we have extensively studied the interaction of the transition metal carboxylates e.g. copper(II), cadmium(II), and zinc(II) benzoates with a variety of spacers such as quinoxaline, 6-methylquinoline, 3-methylquinoline, *trans*-1-(2-pyridyl)-2-(4-pyridyl)ethylene, and di-2-pyridyl ketone (Lee *et al.*, 2008; Yu *et al.*, 2008; Park *et al.*, 2008; Shin *et al.*, 2009; Yu *et al.*, 2009; Yu *et al.*, 2010; Kim *et al.*, 2011). However, nickel as a metal ion source has rarely been used. In this work, we have employed nickel(II) trimethylacetate as a building block and *trans*-1,2-bis(4-pyridyl)ethene as a ligand. We report here on the structure of a new complex poly[tetraqua[*trans*-1,2-bis(4-pyridyl)ethene]nickel(II) dinitrate].

In the crystal structure of the title compound, [Ni(C_12_H_10_N_2_)(H_2_O)_4_] . 2(NO_3_)]_n_, the Ni^II^ ion lies on a crystallographic inversion center with the distorted octahedral coordination sphere comprising four water ligands and two N donors from the *trans*-related 1,2-bis(4-pyridyl)ethene ligands, which also have crystallographic inversion symmetry (Fig. 1). These ligands bridge the Ni^II^ complex units to form a one-dimensional chain structure. The nitrate counter-anions stabilize the crystal structure through water O—H···O_nitrate_ hydrogen bonds (Table 1).

### Experimental

36.4 mg (0.125 mmol) of Ni(NO_3_)_2_^.^6H_2_O and 29.0 mg (0.25 mmol) of (CH_3_)_3_CCOOH and 29.5 mg (0. 25 mmol) of NH_4_OH were dissolved in 4 ml of methanol and carefully layered with 4 ml of a chloroform solution of *trans*-1,2-bis(4-pyridyl)ethene (47.0 mg, 0.25 mmol). Crystals of the title compound suitable for X-ray analysis were obtained within a month.

### Refinement

H atoms were placed in calculated positions with C—H distances of 0.93 Å (pyridyl) and included in the refinement with a riding-motion approximation with *U*_iso_(H) = 1.2*U*_eq_(C). The water H atoms were located in a difference Fourier, and refined isotropically with O—H restraints (0.93 Å).

### Crystal data

**Table:**

[Ni(C_12_H_10_N_2_)(H_2_O)_4_](NO_3_)_2_	*F*(000) = 452
*M**_r_* = 436.99	*D*_x_ = 1.577 Mg m^−^^3^
Monoclinic, *P*2_1_/*n*	Mo *K*α radiation, λ = 0.71073 Å
Hall symbol: -P 2yn	Cell parameters from 1268 reflections
*a* = 7.415 (3) Å	θ = 2.6–23.4°
*b* = 11.426 (4) Å	µ = 1.11 mm^−^^1^
*c* = 10.950 (4) Å	*T* = 293 K
β = 97.307 (7)°	Block, brown
*V* = 920.1 (6) Å^3^	0.15 × 0.08 × 0.08 mm
*Z* = 2	

### Data collection

**Table:**

Bruker SMART CCD area-detector diffractometer	1116 reflections with *I* > 2σ(*I*)
Radiation source: fine-focus sealed tube	*R*_int_ = 0.173
graphite	θ_max_ = 26.0°, θ_min_ = 2.6°
φ and ω scans	*h* = −8→9
4954 measured reflections	*k* = −11→14
1799 independent reflections	*l* = −11→13

### Refinement

**Table:**

Refinement on *F*^2^	Primary atom site location: structure-invariant direct methods
Least-squares matrix: full	Secondary atom site location: difference Fourier map
*R*[*F*^2^ > 2σ(*F*^2^)] = 0.068	Hydrogen site location: inferred from neighbouring sites
*wR*(*F*^2^) = 0.238	H atoms treated by a mixture of independent and constrained refinement
*S* = 1.14	*w* = 1/[σ^2^(*F*_o_^2^) + (0.1257*P*)^2^] where *P* = (*F*_o_^2^ + 2*F*_c_^2^)/3
1799 reflections	(Δ/σ)_max_ < 0.001
136 parameters	Δρ_max_ = 1.08 e Å^−^^3^
4 restraints	Δρ_min_ = −1.86 e Å^−^^3^

### Special details

**Table:**

Geometry. All e.s.d.'s (except the e.s.d. in the dihedral angle between two l.s. planes) are estimated using the full covariance matrix. The cell e.s.d.'s are taken into account individually in the estimation of e.s.d.'s in distances, angles and torsion angles; correlations between e.s.d.'s in cell parameters are only used when they are defined by crystal symmetry. An approximate (isotropic) treatment of cell e.s.d.'s is used for estimating e.s.d.'s involving l.s. planes.
Refinement. Refinement of *F*^2^ against ALL reflections. The weighted *R*-factor *wR* and goodness of fit *S* are based on *F*^2^, conventional *R*-factors *R* are based on *F*, with *F* set to zero for negative *F*^2^. The threshold expression of *F*^2^ > σ(*F*^2^) is used only for calculating *R*-factors(gt) *etc*. and is not relevant to the choice of reflections for refinement. *R*-factors based on *F*^2^ are statistically about twice as large as those based on *F*, and *R*- factors based on ALL data will be even larger.

### Fractional atomic coordinates and isotropic or equivalent isotropic displacement parameters (Å^2^)

**Table:**

	*x*	*y*	*z*	*U*_iso_*/*U*_eq_	
C1	0.0496 (8)	0.7389 (5)	−0.0578 (7)	0.0346 (15)	
H1	−0.0705	0.7450	−0.0939	0.042*	
C2	0.1365 (8)	0.6328 (5)	−0.0637 (7)	0.0414 (18)	
H2	0.0758	0.5697	−0.1038	0.050*	
C3	0.3181 (8)	0.6207 (5)	−0.0086 (7)	0.0364 (16)	
C4	0.3954 (8)	0.7169 (5)	0.0524 (7)	0.0374 (16)	
H4	0.5137	0.7129	0.0923	0.045*	
C5	0.2991 (7)	0.8183 (5)	0.0544 (7)	0.0350 (16)	
H5	0.3542	0.8814	0.0981	0.042*	
C6	0.4117 (9)	0.5087 (5)	−0.0174 (8)	0.0431 (19)	
H6	0.3429	0.4452	−0.0495	0.052*	
N1	0.1284 (6)	0.8326 (4)	−0.0032 (5)	0.0271 (11)	
Ni1	0.0000	1.0000	0.0000	0.0287 (4)	
O1	0.0809 (9)	1.0135 (4)	0.1951 (7)	0.0630 (17)	
H1A	0.098 (12)	0.946 (4)	0.242 (7)	0.076*	
H1B	0.200 (4)	1.036 (7)	0.189 (9)	0.076*	
O2	0.2381 (6)	1.0811 (4)	−0.0466 (7)	0.0666 (18)	
H2A	0.226 (11)	1.148 (5)	−0.095 (7)	0.080*	
H2B	0.306 (10)	1.024 (6)	−0.080 (9)	0.080*	
N2	0.4686 (7)	0.8286 (5)	0.7660 (6)	0.0481 (16)	
O3	0.4935 (7)	0.9318 (4)	0.8009 (6)	0.0684 (17)	
O4	0.5938 (8)	0.7571 (5)	0.7875 (7)	0.0699 (18)	
O5	0.3209 (7)	0.7990 (5)	0.7106 (7)	0.083 (2)	

### Atomic displacement parameters (Å^2^)

**Table:**

	*U*^11^	*U*^22^	*U*^33^	*U*^12^	*U*^13^	*U*^23^
C1	0.031 (3)	0.026 (3)	0.046 (4)	0.005 (2)	0.000 (3)	−0.002 (3)
C2	0.030 (3)	0.023 (3)	0.069 (5)	0.003 (2)	−0.005 (3)	−0.010 (3)
C3	0.030 (3)	0.018 (3)	0.060 (5)	0.009 (2)	−0.001 (3)	0.004 (3)
C4	0.028 (3)	0.018 (3)	0.064 (5)	0.004 (2)	−0.005 (3)	0.000 (3)
C5	0.024 (3)	0.020 (3)	0.060 (5)	0.001 (2)	0.001 (3)	−0.001 (3)
C6	0.037 (3)	0.014 (3)	0.076 (6)	0.009 (2)	0.000 (3)	−0.003 (3)
N1	0.027 (2)	0.022 (2)	0.033 (3)	0.0061 (19)	0.005 (2)	−0.001 (2)
Ni1	0.0214 (6)	0.0136 (6)	0.0485 (8)	0.0038 (4)	−0.0049 (5)	−0.0014 (4)
O1	0.066 (4)	0.051 (3)	0.066 (4)	0.000 (3)	−0.013 (3)	0.000 (3)
O2	0.044 (3)	0.035 (3)	0.121 (6)	0.003 (2)	0.010 (3)	0.012 (3)
N2	0.040 (3)	0.036 (3)	0.064 (5)	−0.003 (2)	−0.012 (3)	−0.010 (3)
O3	0.064 (3)	0.035 (3)	0.098 (5)	−0.008 (2)	−0.018 (3)	−0.016 (3)
O4	0.059 (3)	0.059 (3)	0.091 (5)	0.017 (3)	0.006 (3)	−0.008 (3)
O5	0.055 (3)	0.074 (4)	0.110 (6)	−0.017 (3)	−0.030 (4)	−0.016 (4)

### Geometric parameters (Å, °)

**Table:**

C1—N1	1.326 (7)	N1—Ni1	2.138 (4)
C1—C2	1.378 (8)	Ni1—O2	2.113 (5)
C1—H1	0.9300	Ni1—O2^ii^	2.113 (5)
C2—C3	1.411 (8)	Ni1—N1^ii^	2.138 (4)
C2—H2	0.9300	Ni1—O1^ii^	2.149 (7)
C3—C4	1.373 (8)	Ni1—O1	2.149 (7)
C3—C6	1.465 (8)	O1—H1A	0.93 (6)
C4—C5	1.362 (7)	O1—H1B	0.93 (4)
C4—H4	0.9300	O2—H2A	0.93 (6)
C5—N1	1.350 (7)	O2—H2B	0.93 (7)
C5—H5	0.9300	N2—O5	1.231 (6)
C6—C6^i^	1.331 (13)	N2—O4	1.236 (7)
C6—H6	0.9300	N2—O3	1.245 (7)
			
N1—C1—C2	123.4 (5)	O2^ii^—Ni1—N1^ii^	90.05 (18)
N1—C1—H1	118.3	O2—Ni1—N1	90.05 (18)
C2—C1—H1	118.3	O2^ii^—Ni1—N1	89.95 (18)
C1—C2—C3	119.5 (5)	N1^ii^—Ni1—N1	180.0
C1—C2—H2	120.3	O2—Ni1—O1^ii^	85.9 (3)
C3—C2—H2	120.3	O2^ii^—Ni1—O1^ii^	94.1 (3)
C4—C3—C2	116.5 (5)	N1^ii^—Ni1—O1^ii^	90.7 (2)
C4—C3—C6	124.0 (5)	N1—Ni1—O1^ii^	89.3 (2)
C2—C3—C6	119.5 (5)	O2—Ni1—O1	94.1 (3)
C5—C4—C3	120.1 (5)	O2^ii^—Ni1—O1	85.9 (3)
C5—C4—H4	119.9	N1^ii^—Ni1—O1	89.3 (2)
C3—C4—H4	119.9	N1—Ni1—O1	90.7 (2)
N1—C5—C4	123.9 (6)	O1^ii^—Ni1—O1	179.999 (2)
N1—C5—H5	118.1	Ni1—O1—H1A	119 (6)
C4—C5—H5	118.1	Ni1—O1—H1B	96 (6)
C6^i^—C6—C3	124.7 (7)	H1A—O1—H1B	102 (8)
C6^i^—C6—H6	117.7	Ni1—O2—H2A	118 (5)
C3—C6—H6	117.7	Ni1—O2—H2B	108 (5)
C1—N1—C5	116.5 (5)	H2A—O2—H2B	112 (9)
C1—N1—Ni1	123.9 (4)	O5—N2—O4	120.8 (6)
C5—N1—Ni1	119.6 (4)	O5—N2—O3	120.0 (6)
O2—Ni1—O2^ii^	180.0	O4—N2—O3	119.3 (5)
O2—Ni1—N1^ii^	89.95 (18)		
			
N1—C1—C2—C3	0.7 (12)	C4—C5—N1—C1	4.3 (10)
C1—C2—C3—C4	2.2 (11)	C4—C5—N1—Ni1	−176.6 (6)
C1—C2—C3—C6	−178.6 (7)	C1—N1—Ni1—O2	−134.3 (5)
C2—C3—C4—C5	−1.8 (11)	C5—N1—Ni1—O2	46.7 (5)
C6—C3—C4—C5	179.1 (7)	C1—N1—Ni1—O2^ii^	45.7 (5)
C3—C4—C5—N1	−1.5 (11)	C5—N1—Ni1—O2^ii^	−133.3 (5)
C4—C3—C6—C6^i^	−9.6 (17)	C1—N1—Ni1—O1^ii^	−48.4 (5)
C2—C3—C6—C6^i^	171.3 (11)	C5—N1—Ni1—O1^ii^	132.5 (5)
C2—C1—N1—C5	−3.8 (10)	C1—N1—Ni1—O1	131.6 (5)
C2—C1—N1—Ni1	177.1 (6)	C5—N1—Ni1—O1	−47.5 (5)

Symmetry codes: (i) −*x*+1, −*y*+1, −*z*; (ii) −*x*, −*y*+2, −*z*.

### Hydrogen-bond geometry (Å, °)

**Table:**

*D*—H···*A*	*D*—H	H···*A*	*D*···*A*	*D*—H···*A*
O2—H2B···O3^iii^	0.93 (7)	2.28 (8)	3.176 (9)	162 (8)
O2—H2A···O5^iv^	0.93 (6)	2.14 (7)	3.068 (8)	176 (7)
O1—H1B···O3^v^	0.93 (4)	2.29 (2)	3.212 (9)	170 (8)
O1—H1A···O4^vi^	0.93 (6)	2.37 (3)	3.252 (8)	158 (7)

Symmetry codes: (iii) *x*, *y*, *z*−1; (iv) −*x*+1/2, *y*+1/2, −*z*+1/2; (v) −*x*+1, −*y*+2, −*z*+1; (vi) *x*−1/2, −*y*+3/2, *z*−1/2.

## References

[bb1] Bruker (1997). *SMART and *SAINT** Bruker AXS Inc., Madison, Wisconsin, USA.

[bb2] Daniele, P. G., Foti, C., Gianguzza, A., Prenesti, E. & Sammartano, S. (2008). *Coord. Chem. Rev.* **252**, 1093–1107.

[bb3] Kim, J. H., Kim, C. & Kim, Y. (2011). *Acta Cryst.* E**67**, m3–m4. 10.1107/S1600536810049457 PMC3050134 21522553

[bb4] Lee, E. Y., Park, B. K., Kim, C., Kim, S.-J. & Kim, Y. (2008). *Acta Cryst.* E**64**, m286. 10.1107/S1600536807067876 PMC2960297 21201265

[bb5] Park, B. K., Jang, K.-H., Kim, P.-G., Kim, C. & Kim, Y. (2008). *Acta Cryst.* E**64**, m1141. 10.1107/S1600536808024859 PMC2960550 21201597

[bb6] Parkin, G. (2004). *Chem. Rev.* **104**, 699–767. 10.1021/cr0206263 14871139

[bb7] Sheldrick, G. M. (2008). *Acta Cryst.* A**64**, 112–122. 10.1107/S0108767307043930 18156677

[bb8] Shin, D. H., Han, S.-H., Kim, P.-G., Kim, C. & Kim, Y. (2009). *Acta Cryst* E**65**, m658–m659. 10.1107/S1600536809017772 PMC2969578 21583021

[bb9] Tshuva, E. Y. & Lippard, S. J. (2004). *Chem. Rev.* **104**, 987–1012. 10.1021/cr020622y 14871147

[bb10] Yu, S. M., Koo, K., Kim, P.-G., Kim, C. & Kim, Y. (2010). *Acta Cryst.* E**66**, m61–m62. 10.1107/S1600536809052714 PMC2980161 21579957

[bb11] Yu, S. M., Park, C.-H., Kim, P.-G., Kim, C. & Kim, Y. (2008). *Acta Cryst.* E**64**, m881–m882. 10.1107/S1600536808016516 PMC2961840 21202752

[bb12] Yu, S. M., Shin, D. H., Kim, P.-G., Kim, C. & Kim, Y. (2009). *Acta Cryst.* E**65**, m1045–m1046. 10.1107/S1600536809030281 PMC2969950 21577407

